# Assessing the Feasibility and Acceptability of Implementing a Preclinic Vital Signs Assessment in Primary Care: Cross-Sectional Pilot Study

**DOI:** 10.2196/72655

**Published:** 2026-06-24

**Authors:** John Broughan, Seán McMahon, Steen Gordon, Nandakumar Ravichandran, Donal Bailey, Jennifer Grant, Geoff McCombe, James Sheil, Walter Cullen

**Affiliations:** 1 Clinical Research Centre, School of Medicine, University College Dublin, Health Sciences Centre, Belfield, Dublin 4, Dublin, Ireland, + 353 868928972; 2 Wavescope Ireland Ltd, Dublin, Ireland; 3 School of Medicine, University College Dublin, Dublin, Ireland; 4 Department of Clinical Innovation, Centric Health Primary Care, Dublin, Ireland; 5 Beacon HealthCheck, Beacon Hospital, Dublin, Ireland

**Keywords:** feasibility studies, general practice, pilot projects, primary health care, vital signs

## Abstract

**Background:**

Vital signs are objective measurements of the body’s most basic, essential functions, indicating overall health status. However, such assessments are time-consuming and so are not always prioritized. Measuring vital signs before doctor visits may, therefore, be an effective and efficient strategy.

**Objective:**

We piloted a preclinic vital signs assessment (PCVSA) procedure within a primary care center to determine its feasibility and acceptability.

**Methods:**

A mixed methods cross-sectional design was used for piloting the PCVSA procedure. Study participants included adult patients and practice staff. Patients had vital signs assessed by a primary care assistant before general practitioner (GP) visits. Collected data concerned participants’ study engagement, the timings of PCVSA/GP visits, and surveys/interviews investigating participants’ experiences.

**Implementation (Results):**

A total of 16 patients and 4 staff participated. The mean duration for PCVSAs was 2 minutes and 23 seconds (SD 38.8 s), and the mean duration for GP visits was 9 minutes and 21 seconds (SD 252.4 s). Patients said the PCVSA was a “Positive experience” (n=14, 88%), “Helpful” (n=13, 81%), “Valuable” (n=7, 44%), and “Interesting” (n=6, 38%). The GP said the PCVSAs were either “Helpful” (8/15, 53%) or “Extremely Helpful” (7/15, 47%) in each of their consultations and that the PCVSAs improved engagement with patients (12/15, 80%), allowed them to spend more time gaining an understanding of the conditions of patients (14/15, 93%), and enhanced productivity during consultations (11/15, 73%). The GP strongly agreed that collecting PCVSA data before appointments would benefit patients over time. Qualitative interviews with practice staff yielded three themes: (1) improved patient engagement and efficient consultation, (2) time-saving potential, and (3) practicing in general practice and associated challenges.

**Conclusions:**

The PCVSA pilot showed good feasibility and acceptability as indicated by high participant engagement, short PCVSA and GP visit times (albeit GP visit times did not measure non–patient-facing clinical activity), and positive feedback from patients and staff. Introducing PCVSAs in health care settings may have potential in terms of improving the standard and efficiency of care.

## Introduction

### Context

Assessing patients’ vital signs (VS) is an important health care component, particularly among patients receiving acute care. VS are measurements of the body’s basic functions, indicating overall health. The main VS routinely monitored by health care providers are body temperature, pulse rate, respiration rate, blood pressure, and oxygen saturation (SpO_2_) [1]. VS assessment allows objective investigation and monitoring of patient health. Patient safety is also a concern in health care, and studies report that VS assessment helps improve patient safety in medical units, reducing the risk of adverse events and providing better patient care [1-3]. Thus, regular VS assessment can be important to ensure efficient and potentially critical clinical follow-up and intervention for patients as necessary [4].

Despite the well-established benefits of VS assessments, global health care professional shortages have made VS screening often impractical in busy modern health care settings [5]. Adding VS assessments as they are conducted today to every consultation would be problematic given the pressures on staffing and clinical flow [6]. A survey of 25 general practitioners (GPs) conducted by Wavescope Ltd from February 2023 to January 2024 found that GPs take patients’ VS during 30% of consultations and that they could spend approximately 3 minutes taking VS in each consultation. Based on standard working hours, this indicates that full-time GPs in Ireland spend approximately 110 hours per year measuring patients’ VS.

VS measurements can also result in “white coat hypertension,” a phenomenon of elevated blood pressure due to patient stress in the presence of health care professionals [7]. However, studies consistently show that having blood pressure measured by nonphysician staff such as primary care assistants (PCAs) results in significantly lower readings and less “white coat effect” compared to measurements taken by a physician [8]. Research also indicates that VS assessments are often undervalued by clinicians and not recorded faithfully or frequently enough. For instance, a retrospective analysis by Hayes et al [9] of patients revealed that 98% of patients had substandard VS records.

### Problem Statement

The worst consequence of poor adherence to VS assessment procedures is a greater probability of medical problems deteriorating, potentially to a severe degree among patients with acute health problems [10]. Thus, this study aimed to address these issues by introducing a preclinic VS assessment (PCVSA) procedure in a busy primary health care setting. The study used a Welch Allyn Connex Vital Signs Monitor 6000 Series (MDI Ireland Ltd) [11]. Radio frequency identification (RFID) technology was used to transmit patients’ VS data to the GP via practice management software.

Each patient’s VS assessments were conducted by a PCA while they waited to see the GP. A PCA in Ireland works under the leadership of other health care professionals in primary care to assist and deliver delegated nursing tasks. While common in Irish primary care, PCAs are not part of every setting. PCAs require a Quality and Qualifications Ireland Level 5 qualification for entry to the profession. The patients’ VS data were produced in a report sent to the GP before they saw the patient, allowing them to maximize time spent on clinical decision-making. We hypothesized that the PCVSA would be feasible and acceptable from the perspectives of participating stakeholders, namely, the GP, support staff, and patients.

### Similar Interventions

This study introduces a novel approach to VS assessment in primary care. The study introduces several new elements, including PCA/practice nurse–led PCVSAs, the application of RFID and time stamping technology to calculate time savings, and the automated sending of VS data via practice software before face-to-face consultations for clinical decision-making. To our knowledge, previous studies have not examined the benefits and drawbacks of conducting VS assessments before patients enter the doctor’s office or the seamless integration of these data into doctors’ electronic health records in a primary care setting.

## Methods

### Aims and Objectives

**Figure 1.**
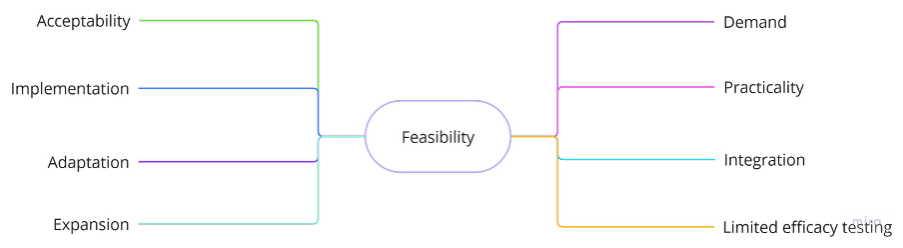
Bowen et al’s [12] feasibility study framework.

We aimed to determine the feasibility and acceptability of implementing a PCVSA procedure in primary care. The feasibility was assessed using Bowen et al’s [12] feasibility framework (Figure 1). This framework helps researchers identify barriers and facilitators, guiding decisions on future studies/trials. Outcome measures included study engagement data, timings of each stage of patients’ GP visits, and self-reported questionnaire and qualitative interview responses from patients and staff regarding their views and experiences of the study and topic (MultimediaAppendices 1  2). These were developed by the research team and informed by the literature. The study is reported according to the iCHECK-DH (Guidelines and Checklist for the Reporting on Digital Health Implementations; Checklist 1) [13].

### Blueprint Summary

Each patient was given an RFID sticker attached to a card [14]. The PCA called each patient for a VS assessment in their office, and patients scanned their RFID card at a VS assessment station. The PCA measured patients’ VS using a VS measurement machine. Provisions were made for up to three VS assessments at 5, 10, and 15 minutes if results were abnormal. The PCA placed the RFID card on the RFID scanner to log the time of examination initiation and termination.

**Figure 2.**
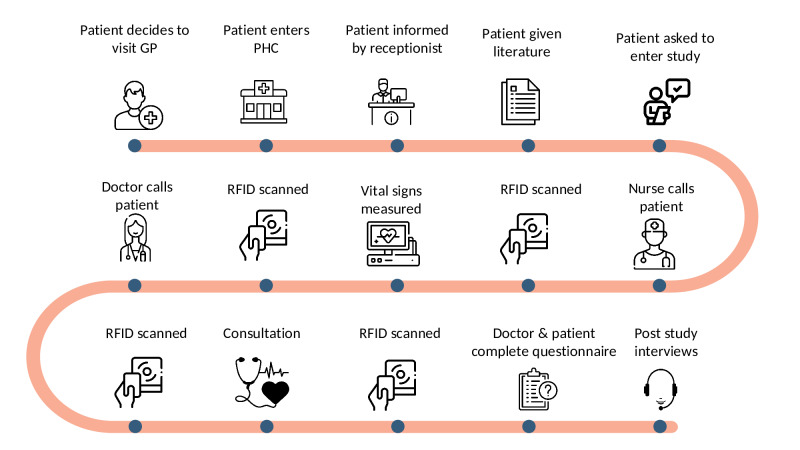
Study procedure. GP: general practitioner; PHC: primary health care center; RFID: radio frequency identification.

Patients returned to the waiting area, and their VS were transmitted to the GP via practice management software for review before the patient was admitted to the GP’s office. Once there, patients scanned their RFID card at the beginning and end of their visit. The GP and patients completed questionnaires regarding their consultation experiences. The GP also completed a doctor satisfaction questionnaire at the study’s end for overall impressions. Qualitative interviews were conducted with staff to investigate their experiences. Figure 2 shows the study procedure.

### Technical Design

The RFID sticker used a featured custom RFID code [15]. This facilitated the collection of RFID data and the time when RFID stickers were presented to RFID readers [16]. As mentioned, the VS measurement machine used was a Welch Allyn Connex Vital Signs Monitor 6000 Series. The practice management software used was Socrates [17].

### Target

The study was conducted in a primary health care center in a Dublin City suburb in Ireland from 9 AM to 12:30 PM each day from July 8 to 10, 2024. Participants included practice staff and a target of 20 adult patients attending GP appointments. Research indicates that our target of 20 was satisfactory [18]. Inclusion and exclusion criteria are in Textbox 1.

Textbox 1. **Inclusion and exclusion criteria.**

**Inclusion criteria**

- Aged >18 years
- Can provide written informed consent to participation (or be supported to provide this as per the Assisted Decision-Making (Capacity) Act 2015)
- Patients due to see the doctor during the study

**Exclusion criteria**

- Patients whom the staff deem too unwell to wait to see a clinician (and are instead being “fast tracked” into assessment)

### Patient Recruitment

Patients were recruited as they attended GP appointments. At check-in, patients were informed by the receptionist that a study was running to evaluate a PCVSA procedure. The patients were given a participant information leaflet (PIL) to read. The PCA and research team assisted patients, who could take home the PIL to think about participating and provide consent during their next visit if the study was still in progress.

### Staff Recruitment

Staff invited to participate were also given a PIL and could discuss the study with the research team.

### Data

#### Measures

Study engagement data were collected by the research team in collaboration with practice staff to determine study recruitment and completion rates. Timings of each stage of patients’ PCA and GP visits were recorded using RFID technology. Patients’ VS data were retained by the clinic, not accessible to the researchers, and not used in the study. Both patients and the GP completed questionnaires following each consultation asking them about their experience. These included patient assessment questionnaires completed by patients, and doctor assessment questionnaires completed by the GP for each patient. Lastly, staff participated in semistructured in-depth qualitative interviews once their involvement was complete. Data were audio recorded, transcribed, and pseudonymized for analysis.

#### Analyses

Descriptive statistics were used to analyze quantitative data. Qualitative coding was used to analyze open response questionnaire data, and analysis of participants’ interviews was guided by Braun and Clarke’s [19] reflexive thematic analysis. Study PILs outlined the data to be collected and stored; procedures for data storage, processing, and sharing; and details about data anonymity. As per open science practices, the study hypotheses and planned analyses were preregistered at AsPredicted [20].

### Participating Entities

The project was led by Wavescope Ltd in partnership with the School of Medicine, University College Dublin (UCD). Wavescope Ltd developed the PCVSA, and UCD led its evaluation. Project funding to Wavescope Ltd was provided by Enterprise Ireland via their Pre-Seed Start Fund [21], and the UCD team was supported by this fund and in-kind contributions from the Health Research Board, Ireland East Hospital Group, and UCD’s College of Health and Agricultural Sciences and School of Medicine. Wavescope Ltd owns the intellectual property of the study’s tracking code and the study design and layout.

### Sustainability

Wavescope is developing a platform that performs VS assessments in waiting rooms using a reusable wearable device, sharing data with clinicians before patients enter the room. Such a system could improve compliance with World Health Organization timing guidelines, reduce white coat hypertension, standardize data collected, reduce time to conduct consultations, and improve patient satisfaction.

### Ethical Considerations

Ethics approval for the study was obtained from the UCD Human Research Ethics Committee (LS-24‐36-Broughan-Cullen). Patients and staff were given a consent form to review and sign before participating. All data were collected, stored, processed, and shared in accordance with the General Data Protection Regulation. A total of €2200 (€1=US $1.15 as of June 12, 2026) was given to support access to clinic facilities and staff time compensation.

## Implementation (Results)

### Coverage

The study was conducted in a primary care center in a Dublin City suburb. The sampling frame (patients scheduled for GP appointments) represents a small part of the patient population in this setting, with 36 patients scheduled (12 daily). Of these, 14 (39%) did not participate because of cancellations, language barriers, or ineligibility. Of the 22 remaining patients, 16 (73%) completed the study. Demographics are presented in Table 1. As per agreements with site management, 4 staff members (GP, PCA, receptionist, practice manager) were invited to participate, and all consented. The GP was male; the others were female.

**Table 1.** Patients’ demographic characteristics.

Demographic characteristics	Partcipants (n=16), n (%)
Gender	
Male	4 (25)
Female	12 (75)
Age group (years)	
18-39	4 (25)
40-64	9 (56)
≥65	3 (19)

### Outcomes

#### PCVSA and GP Visit Timings

Table 2 shows the time spent during the PCVSA and GP interactions.

**Table 2.** Time spent during the preclinic vital signs assessment (PCVSA) and general practitioner (GP) interactions (n=16).

	Time spent (min:s)	
	Mean (SD)	Median (IQR)
PCVSA	2:23 (38.8)	2:36 (108.2‐169.8)
GP visit	9:21 (252.4)	8:59 (364.3‐671.7)

#### Responses to the Patient Assessment Questionnaire

Most of the 16 patients strongly agreed it was a positive experience to take VS before their GP visit (n=14, 88%). All 16 said they would be happy for their GP to collect VS data during every consultation. Thirteen (81%) said the PCVSA was “Helpful,” 7 (44%) said “Valuable,” and 6 (38%) said “Interesting.”

#### GP Responses to the Doctor Assessment Questionnaire

The GP reported that having PCVSA information before meeting patients was “helpful” (8/15, 53%) or “extremely helpful” (7/15, 47%). The GP strongly agreed that the PCVSA improved engagement with more than two-thirds of the patients (12/15, 80%). The GP strongly agreed that PCVSA allowed them to spend more time gaining an understanding of the condition of most of the patients (14/15, 93%). The GP strongly agreed that the PCVSA enhanced productivity gain in the consultations with more than two-thirds of the patients (11/15, 73%).

#### GP Responses to the Doctor Satisfaction Questionnaire

The GP said the PCVSA was beneficial. They strongly agreed that collecting PCVSA data before meeting patients would benefit patients long-term. When asked which VS (temperature, heart rate, respiration, blood pressure, or SpO_2_) were most useful, they said all were equally useful. Additionally, the GP suggested height and weight measures for patients with chronic conditions and electrocardiograms. The GP was uncertain about accepting data from patients’ wearable devices.

#### Qualitative Outcomes

In-depth semistructured qualitative interviews were conducted with 4 staff members (GP, PCA, receptionist, and practice manager). Three themes were identified: (1) improved patient engagement and efficient consultation, (2) time-saving potential, and (3) practicing in general practice and associated challenges. Quotes from interviews illustrating these are in Table 3.

**Table 3.** Quotes from qualitative interviews with staff.

Theme	Quote
Improved patient engagement and efficient consultation	“It’s difficult to say (whether the PCVSA improved patient engagement), it differs case to case but yeah, you get more time and more attention.” (GP^a^) “...they feel they get the extra care, because there’s two health professionals looking after them...” (GP) “...it was great for us and for the patient to know that their vital signs were all fine or if there were any problems before they had gone into the GP...” (PCA^b^)
Time-saving potential	“I think I could have managed two or three GPs seeing patients simultaneously” (PCA) “We saw 16 patients, but I would have liked to see more. Unfortunately, some were children, some faced language barriers, and some needed to take information home to translate. Additionally, some elderly patients could handle limited participation. However, many patients, particularly those in their mid-20s to 40s, were very interested.” (PCA) “Each case is different. Some cases take more time, some cases less...but as a general rule, yeah, the vitals will save time.” (GP)
Practicing in general practice and associated challenges	“...if you have a trauma or anything like that, the vitals are not as important as in acute cases, but even if it’s not acute, there’s no harm in doing it.” (GP) “Most people were happy, but there were some that we found issues with, like, language was a barrier. So, it was challenging to explain the study fully to them... So, they didn’t want to sign-up because they didn’t understand (what it involved).” (Receptionist)

a GP: general practitioner.

b PCA: primary care assistant.

##### Improved Patient Engagement and Efficient Consultation

The GP interviewed was unsure if PCVSA improved patient engagement but reported it allowed more time to understand patients’ conditions and enhance consultation productivity. Checking VS before meeting the GP was seen as providing extra care, with the PCA perceived as a sympathetic, empathetic, and calming support for patients. The PCA reported that knowing the VS assessment results beforehand could allow staff to alert GPs to alarming conditions.

##### Time-Saving Potential

Having VS taken before GP meetings meant that the GP had all the necessary information available, saving time to proceed with patient-facing duties. The PCA reported the intervention being quick and easy, potentially allowing for PCVSA to be implemented across 2 to 3 parallel GPs. This efficiency enabled the GP to meet more than 16 patients per day. While participants were not asked directly about barriers and challenges to the intervention, they did highlight language barriers and participation challenges encountered by older patients. While time savings varied by case, the GP said the initiative saved time.

##### Practicing in General Practice and Associated Challenges

The GP highlighted that the PCVSA would be particularly beneficial for patients with acute conditions. Both patients and staff expressed satisfaction with the implementation of PCVSA in general practice. However, challenges were noted, particularly related to language barriers during consenting and the need for additional staff to perform PCVSA alongside other nursing duties.

### Lessons Learned

#### Success Factors

The study showed good feasibility as evidenced by short durations spent during PCA assessments and GP meetings, as well as good acceptability among patients and staff. Frequently observed positive impacts included a desire to see PCVSA being implemented routinely, perceived improvements in care quality and efficiency, and perceived enhancements regarding staff/patient relationships and communication. Preserving the PCVSA method implemented here for future studies is recommended to ensure that efforts yield the same positive results as was the case here.

#### Challenges to Implementation

While there were good study recruitment and completion rates (16/22, 72%), there were also challenges. Fourteen (39%) patients scheduled for GP appointments were not eligible because they canceled appointments, did not meet eligibility criteria, or were deemed to have insufficient English language skills to comprehend the PIL and consent form. Translated study documentation to overcome language barriers and more participating GPs (and thus more patients) would boost future research’s recruitment numbers. These challenges aside, the most notable issue was the additional staff workload that routine PCVSAs could have. However, the PCA reported that they could have conducted more PCVSAs within the study time frame.

#### Budget

The implementation budget for this study was adhered to. Costs included clinic facilities and staffing (€2200), software development (€10,000), hardware procurement (€314), general development (€3642), and evaluation (€17,804.47).

## Discussion

### Summary of Findings

The PCVSA could be a feasible and acceptable initiative in primary care. The findings suggest that PCVSAs have potential for saving time, enhancing patient engagement, and facilitating more productive and efficient consultations.

### Comparisons With Existing Literature

Previous research indicates that VS assessment can be important to ensure effective clinical evaluation, providing crucial health data for triage, prevention, and safety, particularly for patients who are vulnerable or in the hospital [22-26]. Our study highlights the importance of PCVSAs in a GP setting, with the findings emphasizing the perceived value among practice staff of PCVSAs in saving time and improving patient engagement and consultation quality.

The average time reported by the PCA for conducting PCVSAs (2.4 min) is notably shorter than the 3.75 minutes reported by a similar study [27]. However, that study was conducted in a hospital rather than primary care setting, and research mentions that meaningfully comparing VS assessment timings between studies is difficult because of variations in assessment methods [3]. It is important to note that the average GP visit time reported in this study (9 min and 21 s) only captures the GP’s patient-facing work and should not be confused with the complete consultation time. Previous research by Crosbie et al [28] and Pierse et al [29] calculated this to range from 14.1 minutes to 14 minutes and 36 seconds, and a survey of Irish GPs found the average consultation time in Irish GP practices was 13.7 minutes [30].

Ullah et al [5] reported on the increased workload associated with VS measurement in general practice, an important consideration. However, our study’s findings demonstrate that the use of PCVSAs, despite concerns about staff workload and language barriers during consenting, did not hinder the PCA from managing 16 patients. This led to a perceived reduction in time spent during GP visits and GP workload among participating staff, as well as improved patient experiences and satisfaction. Elliott et al [31] identified gaps in the assessment of VS, emphasizing the need for consistent and thorough measurement to ensure patient safety and effective surveillance in acute settings [31]. Our findings provide support for this position in primary care. The positive feedback from patients and staff underscored PCVSAs’ value in improving patient engagement, consultation efficiency, and overall satisfaction.

### Future Implications

The PCVSAs could have several positive implications for practice. It might lead to improvements in care quality, identifying health problems, operational workflow and time management, patient/staff satisfaction, and patient/staff relationships and communication. A barrier to routinely conducting VS assessments is the extra time and workload assessments may bring [5]. A PCVSA that does not require direct staff involvement (eg, using validated wearable devices) may overcome this. From a research perspective, the main implication of our findings is the need for continued research examining PCVSA’s potential in primary care as well as other contexts. Larger-scale feasibility studies in diverse settings are advised to show proof of concept, and randomized controlled trials may have value in terms of comparing PCVSAs to usual care on key outcomes, perhaps GP visit timings, identification of health problems, or hospital admission frequency.

### Study Limitations

The sample was small and specific to adult patients and staff in one practice, thus limiting generalizability. Testing in larger and more diverse samples is required. GP visits were timed from when patients entered the GP’s office to when they left. Future studies should also include GP time for non–patient-facing duties such as essential administrative, management, and clinical support tasks.

### Conclusions

The study demonstrated good levels of feasibility and acceptability with regard to using a PCA for PCVSAs in terms of saving time and improving patient engagement, perceived consultation efficiency, and satisfaction. However, it also highlighted feasibility and acceptability challenges that need addressing in the future. First, language barriers impacted recruitment, and future studies should take measures to address this. Second, GP visits were timed from entry to exit and excluded time spent performing non–patient-facing duties. The short time frame of this study did not allow for adaptation to address these issues. Furthermore, the study was piloted in a single practice whose staff were motivated to participate, and this may have biased their evaluation. The short duration of the study may have resulted in a limited impact on practice workload compared to a large-scale study. While these factors limit the findings’ generalizability, their findings still suggest that a PCVSA procedure would be both feasible and acceptable.

## Supplementary material

10.2196/72655 Multimedia Appendix 1 Quantitative questionnaires.

10.2196/72655 Multimedia Appendix 2 Qualitative interview topic guide.

10.2196/72655 Checklist 1 iCHECK-DH checklist.

## Abbreviations

GP: general practitioner
iCHECK-DH: Guidelines and Checklist for the Reporting on Digital Health Implementations
PCA: primary care assistant
PCVSA: preclinic vital signs assessment
PIL: participant information leaflet
RFID: radio frequency identification
SpO_2_: oxygen saturation
UCD: University College Dublin
VS: vital signs
