# Supplementary material for: Assessing the Feasibility and Acceptability of Implementing a Preclinic Vital Signs Assessment in Primary Care: Cross-Sectional Pilot Study
Source: JMIR Med Inform. 2026 Jun 24;14:e72655. doi: 10.2196/72655 (PMC13293563; doi:10.2196/72655)
Supplement: Multimedia Appendix 1 [file medinform-v14-e72655-s001.pdf]

Appendix 1 Quantitative questionnaires

Doctor Assessment Questionnaire

What was the value of having the Pre-Clinic Vital Signs Assessment (PCVSA) information at the start of the visit?

Unhelpful	No impact at all	Helpful	Extremely Helpful

Did having the vital signs assessment prior to this visit…

	Strongly Agree	Disagree	Neutral	Agree	Strongly Agree
1. Improve how you engaged with the patient	1	2	3	4	5
2. Allow you spend more time gaining understanding of the patient's condition	1	2	3	4	5
3. Offer any time or productivity gain in the consultation	1	2	3	4	5

Can you elaborate on any other perceived benefit?

____

____

____

____

____

____

____

____

Doctor satisfaction questionnaire

This is to be completed when the pilot study concludes.

Overall, did you find it beneficial to have patient vital signs data in advance of their visit?

Yes ☐ No ☐

Do you think that collating serial vital signs assessment will have benefit your patients in the long run?

Strongly Agree	Disagree	Neutral	Agree	Strongly Agree
1	2	3	4	5

Which (if any) Vital Sign(s) did you find to be the most useful. (please rank)

Temperature ☐

Heart Rate ☐

Respiration Rate ☐

Blood Pressure ☐

Oxygen Saturation (SpO2) ☐

Would you like other measurement(s) to be taken in advance of your clinic such as height, weight, body fat, muscle mass analysis, digital ECG?

1. ____
2. ____
3. ____

Would you accept vital signs data shared with you by a patient if it came from their own wearable device?

Yes ☐ No ☐

If you answered no to the above, please briefly elaborate on why.

____

Patient assessment questionnaire

Having my vital signs assessment taken prior to meeting the GP was a positive experience.

Strongly Agree	Disagree	Neutral	Agree	Strongly Agree
1	2	3	4	5

I would be happy for my GP to collect serial data on my vital signs during every future consultation.

Yes ☐ No ☐

Did you find having a pre-clinical vital signs assessment today was:

Please tick all that apply

Helpful ☐ Valuable ☐ Interesting ☐

Any other comment on the experience?

____

____

____

____

____

____

____

____
