# Supplementary material for: Assessing the Feasibility and Acceptability of Implementing a Preclinic Vital Signs Assessment in Primary Care: Cross-Sectional Pilot Study
Source: JMIR Med Inform. 2026 Jun 24;14:e72655. doi: 10.2196/72655 (PMC13293563; doi:10.2196/72655)
Supplement: Multimedia Appendix 2 [file medinform-v14-e72655-s002.pdf]

Appendix 2 Qualitative interview topic guide

Qualitative interview topic guide

a) How was your experience of this study?

- What did you like about it? What did you dislike about it? Was it helpful? Valuable? Interesting? Why was this the case?

b) Have you ever done something like this (i.e., pre-clinic assessment) in your practice (or in other settings) before? If yes, how did your experience of this study compare with that/those prior experience(s)?

c) Did the pre-clinic vital signs assessment help you:

- Improve how you engaged with the patient? If yes, how so?
- Spend more time gaining understanding of the patient's condition? If yes, how so?
- Be more productive or efficient? If yes, how?

d) Do you think there is a need for initiatives like this (pre-clinic vital signs assessment) in primary care and general practice going forward?

- Would you recommend conducting pre-clinic vital signs assessments to primary care/GP colleagues going forward?
- Have you any other feedback you would like to give?
